# Typical antipsychotics is associated with increased risk of severe exacerbation in asthma patients: a nationwide population-based cohort study

**DOI:** 10.1186/s12890-022-01883-6

**Published:** 2022-03-14

**Authors:** Chin-Wei Kuo, Szu-Chun Yang, Yu-Fen Shih, Xin-Min Liao, Sheng-Hsiang Lin

**Affiliations:** 1 Institute of Clinical Medicine, College of Medicine, National Cheng Kung University, Tainan, Taiwan; 2 Department of Internal Medicine, National Cheng Kung University Hospital, College of Medicine, National Cheng Kung University, Tainan, Taiwan; 3 Department of Pharmacy, National Cheng Kung University Hospital, College of Medicine, National Cheng Kung University, Tainan, Taiwan; 4 Department of Public Health, College of Medicine, National Cheng Kung University, Tainan, Taiwan; 5 Biostatistics Consulting Center, National Cheng Kung University Hospital, College of Medicine, National Cheng Kung University, Tainan, Taiwan

**Keywords:** Asthma, Exacerbation, Antipsychotics, Psychiatric disorder

## Abstract

**Background:**

Severe asthma exacerbation reduces patients’ quality of life, results in visits to the emergency department (ED) and hospitalization, and incurs additional medical costs. Antipsychotics block receptors with bronchodilation function; however, the association between antipsychotic use and severe asthma exacerbation is unknown. This study aimed to investigate the effects of antipsychotics on asthma-related ED visits and hospitalizations.

**Methods:**

A case-crossover design was used in this study. Using the 2003–2017 Taiwan National Health Insurance Reimbursement Database, we established a cohort of 18,657 adults with asthma exacerbation leading to ED visits or hospitalization. Univariate and multivariate conditional logistic regressions were conducted to explore the association between antipsychotic use and severe asthma exacerbation. Subgroup analyses of different classes, doses, receptor functions of antipsychotics, different psychiatric disease, and sensitivity analyses of excluding patients with schizophrenia were also performed.

**Results:**

Antipsychotic use was associated with a higher risk of severe asthma exacerbation (adjusted odds ratio [OR]: 1.27; 95% confidence interval [CI] 1.05–1.54; *P* = 0.013) compared with no use of antipsychotics. The use of typical antipsychotics increased the risk of severe asthma exacerbation (adjusted OR: 1.40, 95% CI 1.10–1.79, *P* = 0.007), whereas the use of atypical antipsychotics did not. These results did not change after the exclusion of patients with schizophrenia. There was a dose-dependent effect of antipsychotics (trend test, *P* = 0.025). Antipsychotics that block the M2 muscarinic or D2 dopaminergic receptors were associated with an increased risk of severe asthma exacerbation (adjusted OR: 1.39, 95% CI 1.10–1.76, *P* = 0.007 and adjusted OR: 1.33, 95% CI 1.08–1.63, *P* = 0.008, respectively). However, use of antipsychotics did not increase risk of severe asthma exacerbation in patients with psychiatric disorder.

**Conclusions:**

The use of typical antipsychotics is associated with a dose-dependent increased risk of severe asthma exacerbation, especially for patients without psychiatric disorders. Further research on the impact of typical antipsychotics on asthma exacerbation is warranted.

**Supplementary Information:**

The online version contains supplementary material available at 10.1186/s12890-022-01883-6.

## Background

Asthma is a prevalent airway disease characterized by variable respiratory symptoms and airflow limitations [1]. It is the second most common chronic respiratory disease globally, accounting for 0.88% of the all-cause mortality in 2017 [2]. Exacerbation of asthma is defined as an episodic and progressive increase in asthma-associated respiratory symptoms, which impair patients’ health-related quality of life [3]. Severe exacerbation of asthma leading to emergency department (ED) visits or hospitalization causes human productivity loss and incurs additional medical costs [4]. Moreover, it increases the risk of future exacerbations [5].

Prevalence of antipsychotics use was in 3.5% of Taiwan [6]. In addition to treating schizophrenic disorders, antipsychotics are administered for a variety of psychiatric disorders, such as mood disorders, agitation, delirium, and insomnia [7, 8]. Off-label prescriptions of antipsychotics account for 40–75% of all adult prescriptions [9]. Antipsychotics can be classified into typical and atypical based on their affinity to the D2 dopaminergic receptor and the serotonin 5-HT2A receptor, and the side effects are different between the two groups of drugs owing to their affinity to different receptors [10]. In addition to dopaminergic and serotonin receptors, antipsychotics are multipotent drugs that block several neurotransmitter receptors [10], including the M2 muscarinic and β_2_ adrenergic receptors [11]. The blocking of bronchodilation receptors, such as β_2_ adrenergic receptors, may be associated with acute asthma exacerbation [12]. Crane et al. found a higher risk of asthma-related death and hospital readmission for antipsychotic users [13]. However, this case–control study only analyzed a small number of participants receiving psychotropic drugs, and the enrolled population was limited to individuals aged 5–45 years in New Zealand during 1981–1987. Most atypical antipsychotic drugs that were used clinically received approval from the Food and Drug Administration after the 1990s [14]. The risk of severe asthma exacerbation with the administration of atypical antipsychotics remains unclear. We hypothesized that the use of antipsychotics is associated with an increase in severe asthma exacerbation, leading to ED visits or hospitalization. Using nationwide claims data, we conducted a case-crossover study to validate our hypothesis. We also tested the dose-dependent effect and performed subgroup analyses of different classes and receptor functions of antipsychotics.

## Methods

### Study setting and design

This study used the 2003–2017 Taiwan National Health Insurance Reimbursement Database (NHIRD), which was derived from the Taiwan Health and Welfare Data Science Center. The database includes administrative data of 23 million individuals and covers 98% of the residents in Taiwan [15]. This study used a case-crossover design. We compared the use of antipsychotics and other clinical factors during the period immediately before severe asthma exacerbation with that during an earlier control period. The Institutional Review Board of the National Cheng Kung University Hospital approved this study before commencement (B-EX-109-026). Informed consent was waived due to the use of de-identified information.

### Definition of patients with asthma and severe asthma exacerbation

**Fig. 1:**
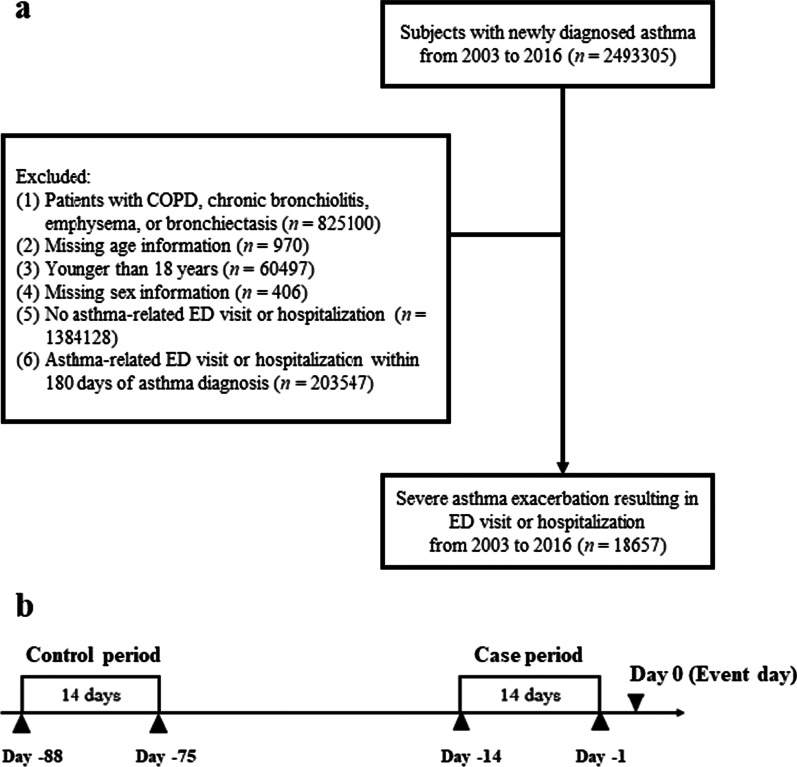
Algorithm for subject enrollment and timeline for case crossover study. **a** Inclusion algorithm for severe asthma exacerbation; **b** timeline for the case and control periods. COPD, chronic obstructive pulmonary disease; ED, emergency department

All patients newly diagnosed with asthma between January 1, 2003, and December 31, 2016, were identified (Fig. 1a). We defined patients with asthma as those who had more than one inpatient or two outpatient visits within 1 year using the *International Classification of Diseases, Ninth Revision* (ICD-9) diagnosis codes 493.xx or ICD-10-CM (Clinical Manifestation) codes J45.x [15]. The accuracy of diagnoses recorded in the NHIRD for asthma has been validated [16]. The first visit was defined as the day of the asthma diagnosis. We excluded patients with a diagnosis of asthma between January 1, 2000, and December 31, 2002, to ensure that all the cases were newly diagnosed. Patients with ICD-9 or ICD-10 codes for chronic obstructive pulmonary disease, chronic bronchiolitis, emphysema, and bronchiectasis, those younger than 18 years, and those who lacked gender information were also excluded. Every patient had at least one asthma-related ED visit or hospitalization during the study period.

Severe asthma exacerbation was defined as an acute exacerbation of asthma resulting in ED visits or hospitalization in combination with the use of short-acting bronchodilators and systemic steroids. To avoid being confounded by previous severe exacerbations, we selected the first episode for analysis. ED visits and hospitalization within 180 days of the day of asthma diagnosis were excluded to avoid confounding from inadequate disease-control-related acute exacerbation at asthma diagnosis.

### Case and control periods

Based on previous studies and the elimination half-lives of antipsychotics (Additional file 1: Table S1) [17, 18], we defined the case and control periods as 1–14 days and 75–88 days, respectively, before severe asthma exacerbation (Fig. 1b). These definitions ensured the washout of drug effects. Patients’ comorbidities during the 180 days before the case period were compared with those during the 180 days before the control period to derive the odds ratios (ORs). The use of other medications during the case period was compared to that during the control period. The ICD codes for comorbidities and medications are listed in Additional file 1: Table S2 and Additional file 1: Table S3.

### Statistical analysis

To accommodate the case-crossover design, we used conditional logistic regression analyses to deal with the matched pair data. We first conducted a univariable analysis to derive the crude OR of each covariate in the case period versus the control period. Covariates with significant crude ORs (*P* < 0.05) in the univariate analysis were used in the multivariate analysis to obtain adjusted ORs. Because of psychiatric disorder affect the management of asthma [19], covariates such like depression, bipolar and schizophrenia were also added in the multivariate analysis. To test the robustness of the results, we performed several sensitivity analyses. First, we categorized patients with asthma using typical antipsychotics, atypical antipsychotics, or both. To compare antipsychotics with other psychiatric drugs, we also classified patients using serotonin–norepinephrine reuptake inhibitor (SNRI), selective serotonin reuptake inhibitors (SSRI) or tricyclic antidepressants (TCA). Second, we examined the dose-dependent effect of antipsychotics by dividing the study cohort into low-dose and medium- to high-dose groups. Both groups were defined based on the defined daily dose (DDD) [20]. A low dose denoted a dose of less than or equal to 0.25 DDD and a medium to high dose denoted a dose of more than 0.25 DDD [17]. Cochran-Armitage trend test was used to confirm whether antipsychotics have a dose-dependent effect on severe asthma exacerbation. Third, schizophrenia was the main indication for antipsychotics. We performed the analysis after excluding enrolled patients with schizophrenia to avoid confounding effects of schizophrenia. Fourth, we classified each antipsychotic by its antagonistic property on different receptors with the bronchial relaxation function, including the β2 adrenergic, M2 muscarinic, D1 dopaminergic, and D2 dopaminergic receptors [21–24]. We defined Ki value < 100 as higher affinity and Ki value > 100 as lower affinity (Additional file 1: Table S4) [11]. Fifth, to avoid confounds due to severe mental illness associated with poor asthma control, we performed a sensitivity analysis excluding patients with at least one depression, bipolar disorder, or schizophrenia-related admissions or emergency department visits within 180 days before the case and control periods. Lastly, we performed subgroup analyses to investigate the effect of antipsychotics on severe asthma exacerbation for patients with schizophrenia, depression and bipolar disorder. All analyses were performed using SAS software (Version 9.4; SAS Institute, Cary NC, USA), and *P-values* were based on two-tailed tests.

## Results

A total of 18,657 patients from 2003 to 2016 with severe asthma exacerbation resulting in ED visits or hospitalization in combination with the use of short-acting bronchodilators and systemic steroids were included in the analysis. Demographic characteristics, comorbidities, and drug exposure during the case and control periods are shown in Table 1. Most of the patients were women. The mean age was 47.7 years on the event day. Enrolled patients were more likely to have more diagnoses of comorbidities, schizophrenia, pneumonia, and acute upper airway infection during the study period. Moreover, enrolled patients were more likely to use antiplatelet drugs, β-blockers, non-steroidal anti-inflammatory drugs (NSAIDs), antidepressants, antipsychotics, anticholinergic agents, antihistamines, inhaled bronchodilators, and oral bronchodilators in the case period than in the control period (Table 1).

**Table 1:** Characteristics of patients in the case and control periods

Characteristics	Number (%)		Conditional logistic regression	
	Case period (*n* = 18,657)	Control period (*n* = 18,657)	Crude OR (95% CI)	*P* value
Male	5577 (29.9)	5577 (29.9)	–	–
Age, mean (SD)	47.7 (17.9)	47.5 (17.7)		
Comorbidities				
Heart failure	395 (2.1)	349 (1.9)	2.18 (1.49–3.18)	< 0.001
Ischemic heart disease	931 (5.0)	873 (4.7)	1.62 (1.25–2.10)	< 0.001
Stroke	530 (2.8)	498 (2.7)	1.62 (1.14–2.28)	0.007
GERD	762 (4.1)	671 (3.6)	1.65 (1.34–2.04)	< 0.001
Obesity	77 (0.4)	49 (0.3)	4.11 (1.98–8.52)	< 0.001
Rhinosinusitis	3393 (18.2)	3125 (16.8)	1.44 (1.30–1.60)	< 0.001
Obstructive sleep apnea	51 (0.3)	44 (0.2)	1.64 (0.77–3.46)	0.198
Psychiatric disorder				
Anxiety	166 (0.9)	159 (0.9)	1.21 (0.76–1.92)	0.415
Bipolar disorder	154 (0.8)	146 (0.8)	1.62 (0.81–3.23)	0.174
Depression	747 (4.0)	741 (4.0)	1.08 (0.79–1.47)	0.633
Schizophrenia	148 (0.8)	136 (0.7)	5.00 (1.45–17.27)	0.011
Respiratory infection				
Pneumonia	726 (3.9)	533 (2.9)	2.44 (2.00–2.98)	< 0.001
Acute upper airway infection	8308 (44.5)	7140 (38.3)	2.00 (1.86–2.14)	< 0.001
Medications				
Cardiovascular drugs				
Antiplatelet	1184 (6.4)	1019 (5.5)	1.52 (1.32–1.75)	< 0.001
Cardioselective β-blockers	716 (3.8)	660 (3.5)	1.24 (1.04–1.47)	0.015
Non-selective β-blockers	676 (3.6)	553 (3.0)	1.52 (1.29–1.78)	< 0.001
Statins	909 (4.9)	883 (4.7)	1.08 (0.93–1.25)	0.324
Anti-inflammation				
Colchicine	138 (0.7)	147 (0.8)	0.90 (0.67–1.22)	0.492
NSAIDs	5175 (27.7)	3098 (16.6)	2.32 (2.19–2.47)	< 0.001
Metformin	753 (4.0)	721 (3.9)	1.15 (0.96–1.39)	0.133
Psychoactive drugs				
Antidepressants	957 (5.1)	857 (4.6)	1.39 (1.19–1.64)	< 0.001
Lithium	16 (0.1)	13 (0.1)	2.00 (0.50–8.00)	0.327
Antipsychotics	713 (3.8)	601 (3.2)	1.48 (1.25–1.74)	< 0.001
Anticholinergic agents				
Bladder	116 (0.6)	93 (0.5)	1.43 (1.01–2.04)	0.044
Gastrointestinal tract	1442 (7.7)	888 (4.8)	1.86 (1.69–2.05)	< 0.001
Parkinsonism	107 (0.6)	100 (0.5)	1.28 (0.76–2.16)	0.358
Antihistamine				
First generation	3234 (17.3)	1817 (9.7)	2.26 (2.11–2.43)	< 0.001
Second/third generation	4372 (23.4)	2615 (14.0)	2.27 (2.13–2.42)	< 0.001
Inhaled bronchodilators				
SABA	2615 (14.0)	1087 (5.8)	3.25 (2.98–3.54)	< 0.001
SAMA	305 (1.6)	60 (0.3)	6.56 (4.78–9.01)	< 0.001
SABA + SAMA	154 (0.8)	79 (0.4)	2.50 (1.80–3.47)	< 0.001
LABA	3 (0.02)	0 (0.0)	–	–
LAMA	37 (0.2)	30 (0.2)	1.44 (0.76–2.72)	0.265
LABA + ICS	1575 (8.4)	1217 (6.5)	1.55 (1.41–1.72)	< 0.001
LABA + LAMA + ICS	0 (0.0)	0 (0.0)	–	–
ICS	381 (2.0)	283 (1.5)	1.50 (1.25–1.80)	< 0.001
Oral bronchodilators				
Xanthium	3844 (20.6)	1771 (9.5)	3.40 (3.15–3.66)	< 0.001
Leukotriene receptor antagonists	550 (3.0)	404 (2.2)	1.78 (1.49–2.12)	< 0.001

GERD: gastroesophageal reflux disease; ICS: inhaled corticosteroid, LABA: long-acting β-agonist; LAMA: long-acting muscarinic antagonist; NSAID: non-steroidal anti-inflammatory drug; OR: odds ratio; SABA: short-acting β-agonist; SAMA: short-acting muscarinic antagonist

Table 2 shows the results of the conditional logistic regression for the risk of severe asthma exacerbation associated with the use of antipsychotics. There were 713 (3.8%) patients who used antipsychotics during the case period and 601 (3.2%) patients during the control period. The adjusted OR was 1.27 (95% confidence interval [CI], 1.05–1.54; *P* = 0.013). The adjusted ORs of all covariates in the multivariable conditional logistic regression analysis are shown in Additional file 1: Table S5. Schizophrenia increased the risk of severe asthma exacerbation (adjusted OR, 5.46; 95% CI 1.48–20.18, *P* = 0.011). For patients with schizophrenia, depression or bipolar disorder, use of antipsychotics did not increase the risk of asthma severe exacerbation (Additional file 1: Table S6, Additional file 1: Table S7 and Additional file 1: Table S8). After excluding patients with schizophrenia, any use of antipsychotics increased the risk of severe asthma exacerbation (adjusted OR, 1.29; 95% CI 1.07–1.57, *P* = 0.009) (Table 3). Similarly, any use of antipsychotics increased the risk of severe asthma exacerbation after excluding patients with psychiatric disorder-related ER visits or hospitalizations (adjusted OR: 1.29, 95% CI 1.06–1.56, *P* = 0.009) (Additional file 1: Table S9). On the other hand, use of antidepressant did not increase the risk of severe asthma exacerbation (Additional file 1: Table S10).

**Table 2:** Risk of severe asthma exacerbation by different classes of antipsychotics and doses

Characteristics	No. (%)		Conditional logistic regression				P for trend
	Case period (n = 18,657)	Control period (n = 18,657)	Crude OR (95% CI)	P value	Adjusted OR^a^ (95% CI)	P value	
No use of anti-psychotics	17,944 (96.18)	18,056 (96.78)	Ref		Ref		
Any use of anti-psychotics	713 (3.82)	601 (3.22)	1.48 (1.25–1.74)	< 0.001	1.27 (1.05–1.53)	0.015	
Antipsychotics class^b^							
Typical only	269 (1.44)	193 (1.03)	1.63 (1.31–2.04)	< 0.001	1.42 (1.10–1.82)	0.006	
Atypical only	411 (2.20)	390 (2.09)	1.25 (0.98–1.59)	0.072	1.05 (0.80–1.38)	0.704	
Both	33 (0.18)	18 (0.10)	3.03 (1.40–6.57)	0.005	2.34 (1.02–5.35)	0.045	
Dose							
Low (≤ 0.25 DDD)	604 (3.24)	504 (2.70)	1.47 (1.24–1.74)	< 0.001	1.25 (1.03–1.50)	0.023	0.038
Medium to high (> 0.25 DDD)	109 (3.24)	97 (0.52)	1.57 (1.11–2.21)	0.011	1.47 (1.00–2.15)	0.049	
Dose (for typical only)							
Low (≤ 0.25 DDD)	255 (1.37)	184 (0.99)	1.61 (1.28–2.02)	< 0.001	1.37 (1.06–1.75)	0.015	0.006
Medium to high (> 0.25 DDD)	14 (0.08)	9 (0.05)	2.85 (0.96–8.50)	0.060	4.58 (1.36–15.40)	0.014	
Dose (for atypical only)							
Low (≤ 0.25 DDD)	326 (1.75)	311 (1.67)	1.27 (0.99–1.63)	0.066	1.06 (0.80–1.41)	0.682	0.767
Medium to high (> 0.25 DDD)	85 (0.46)	79 (0.42)	1.36 (0.91–2.03)	0.134	1.18 (0.76–1.83)	0.468	

DDD: defined daily dose; OR: odds ratio

^a^Adjusted for heart failure, ischemic heart disease, stroke, gastroesophageal reflux disease, obesity disorder, rhinosinusitis, schizophrenia, depression, bipolar disorder, pneumonia, acute upper airway infection, antiplatelet agents, cardioselective β-blocker, non-selective β-blocker, NSAID, anti-psychotics, anti-depressants, bladder anticholinergic agents, gastrointestinal tract anticholinergic agents, first generation anti-histamine, second/third generation anti-histamine, short-acting beta-agonist, short-acting muscarinic antagonist, short-acting beta-agonist plus short-acting muscarinic antagonist, long-acting beta-agonist plus long-acting beta-agonist, long-acting beta-agonist, xanthine inhibitor, leukotriene receptor antagonist

^b^See Additional file 1: Table S1

**Table 3:** Risk of severe asthma exacerbation by different classes of antipsychotics and doses after excluding schizophrenia patients

Characteristics	No. (%)		Conditional logistic regression				P for trend
	Case period	Control period	Crude OR (95% CI)	P value	Adjusted OR^a^ (95% CI)	P value	
No use of antipsychotics	17,900 (96.73)	18,008 (97.31)	Ref		Ref		
Any use of antipsychotics	606 (3.3)	498 (2.7)	1.49 (1.26–1.77)	< 0.001	1.28 (1.06–1.57)	0.011	
Antipsychotics class^b^							
Typical only	260 (1.40)	186 (1.01)	1.62 (1.29–2.03)	< 0.001	1.37 (1.07–1.76)	0.012	
Atypical only	326 (1.76)	298 (1.61)	1.32 (1.01–1.71)	0.039	1.15 (0.86–1.54)	0.344	
Both	20 (0.11)	14 (0.08)	1.87 (0.81–4.30)	0.142	1.60 (0.66–3.92)	0.300	
Dose							
Low (≤ 0.25 DDD)	535 (2.89)	441 (2.38)	1.48 (1.25–1.76)	< 0.001	1.27 (1.05–1.54)	0.016	0.005
Medium to high (> 0.25 DDD)	71 (0.38)	57 (0.31)	1.81 (1.17–2.78)	0.007	1.65 (1.03–2.62)	0.036	
Dose (typical only)							
Low (≤ 0.25 DDD)	249 (1.35)	181 (0.98)	1.60 (1.27–2.01)	< 0.001	1.37 (1.06–1.76)	0.015	0.004
Medium to high (> 0.25 DDD)	11 (0.06)	5 (0.03)	4.30 (1.15–16.15)	0.031	5.74 (1.33–24.67)	0.019	
Dose (atypical only)							
Low (≤ 0.25 DDD)	271 (1.46)	252 (1.36)	1.30 (0.99–1.70)	0.057	1.12 (0.83–1.52)	0.451	0.282
Medium to high (> 0.25 DDD)	55 (0.30)	46 (0.25)	1.51 (0.92–2.47)	0.107	1.33 (0.78–2.27)	0.301	

DDD: defined daily dose, OR: odds ratio

^a^Adjusted for heart failure, ischemic heart disease, stroke, gastroesophageal reflux disease, obesity disorder, rhinosinusitis, schizophrenia, depression, bipolar disorder, pneumonia, acute upper airway infection, antiplatelet agents, cardioselective β-blocker, non-selective β-blocker, NSAID, anti-psychotics, anti-depressants, bladder anticholinergic agents, gastrointestinal tract anticholinergic agents, first generation anti-histamine, second/third generation anti-histamine, short-acting beta-agonist, short-acting muscarinic antagonist, short-acting beta-agonist plus short-acting muscarinic antagonist, long-acting beta-agonist plus long-acting beta-agonist, long-acting beta-agonist, xanthine inhibitor, leukotriene receptor antagonist

^b^See Additional file 1: Table S1

The risk of severe asthma exacerbation by different classes of antipsychotics and doses is shown in the lower part of Table 2. The use of typical antipsychotics was associated with an increased risk of severe asthma exacerbation (adjusted OR, 1.40; 95% CI 1.10–1.79, *P* = 0.007). In contrast, the use of atypical antipsychotics did not increase the risk (adjusted OR: 1.10, 95% CI 0.84–1.44, *P* = 0.481). Use of both typical and atypical antipsychotics had a higher risk of asthma exacerbation than the use of one of them (adjusted OR: 2.47, 95% CI 1.09–5.62, *P* = 0.031). A dose-dependent effect was also observed with the use of any class of antipsychotics. The effect was obvious for the use of typical antipsychotics; however, not statistically significant for atypical antipsychotics (in the test for trend, any class of antipsychotics: *P* = 0.025, typical antipsychotics: *P* = 0.006; atypical antipsychotics: *P* = 0.652). After excluding patients with schizophrenia, the results of the analysis were similar to the results of the analysis for all enrolled patients. The use of typical antipsychotics was still associated with an increased risk of severe asthma exacerbation with a dose-dependent effect; however, the use of atypical antipsychotics was not (for typical antipsychotics: adjusted OR: 1.37, 95% CI 1.07–1.76, *P* = 0.012; for atypical antipsychotics: adjusted OR: 1.17, 95% CI 0.88–1.56, *P* = 0.293) (Table 3). Additionally, after excluding patients with psychiatric disorders related to ER visits or hospitalizations, the use of typical antipsychotics increased the risk of severe asthma exacerbation in a dose-dependent manner (adjusted OR: 1.39, 95% CI 1.08–1.78, *P* = 0.010) (Additional file 1: Table S7). For different classes of antidepressants, use of SSRI/SNRI or TCA did not increase the risk of severe asthma exacerbation (Additional file 1: Table S10).

Table 4 shows the risk of severe asthma exacerbation stratified by the different receptor functions of antipsychotics. Agents that block the M2 muscarinic receptor and the D2 dopaminergic receptor are associated with an increased risk of severe asthma exacerbation (adjusted OR: 1.39, 95% CI 1.10–1.76, *P* = 0.007 and adjusted OR: 1.33, 95% CI 1.08–1.63, *P* = 0.008, respectively).

**Table 4:** Risk of severe asthma exacerbation of antipsychotics with high blocking affinity of receptor

Characteristics	Number (%)		Conditional logistic regression			*P* value
	Case period	Control period	Crude OR (95% CI)	*P* value	Adjusted OR^a^ (95% CI)	
No antipsychotics use	17,927	18,037	Ref		Ref	
Antipsychotics with high blocking affinity^b,c^						
β2 adrenergic receptor	302 (1.7)	276 (1.5)	1.37 (0.98–1.92)	0.064	1.25 (0.87–1.81)	0.227
M2 muscarinic receptor	442 (2.4)	356 (1.9)	1.60 (1.29–1.99)	< 0.001	1.39 (1.10–1.76)	0.007
D1 dopaminergic receptor	391 (2.1)	357 (1.9)	1.34 (1.04–1.73)	0.025	1.17 (0.88–1.55)	0.283
D2 dopaminergic receptor	535 (2.9)	440 (2.4)	1.52 (1.26–1.84)	< 0.001	1.33 (1.08–1.63)	0.008

^a^Adjusted for heart failure, ischemic heart disease, stroke, gastroesophageal reflux disease, obesity disorder, rhinosinusitis, schizophrenia, pneumonia, acute upper airway infection, antiplatelet agents, cardioselective β-blocker, non-selective β-blocker, NSAID, anti-psychotics, anti-depressants, bladder anticholinergic agents, gastrointestinal tract anticholinergic agents, first generation anti-histamine, second/third generation anti-histamine, short-acting beta-agonist, short-acting muscarinic antagonist, short-acting beta-agonist plus short-acting muscarinic antagonist, long-acting beta-agonist plus long-acting beta-agonist, long-acting beta-agonist, xanthine inhibitor, leukotriene receptor antagonist

^b^High blocking affinity is defined as Ki value < 100 (see Additional file 1: Table S4)

^c^Antipsychotics without Ki (inhibitory constant) value were excluded from analysis

## Discussion

Although previous studies have shown that the use of antipsychotics at the time of hospital admission increases the risk of asthma-related death and hospital readmission [13], the association between the use of antipsychotics and severe asthma exacerbation has not been investigated in a nationwide asthma population. The effects of atypical antipsychotics on severe asthma exacerbation have not yet been examined. In this case-crossover study, we analyzed 18,657 newly diagnosed asthma patients with severe exacerbation leading to ED visits or hospitalization. Using multivariable conditional logistic regression, we found that the use of antipsychotics was associated with an increased risk of severe asthma exacerbation (adjusted OR: 1.27). This result was not confounded by respiratory infection, schizophrenia, use of NSAIDs or non-selective β-blockers, or different types of inhaled bronchodilator prescriptions. In the subgroup analysis, the use of typical antipsychotics significantly increased the risk of severe asthma exacerbation by 40%. Furthermore, antipsychotics, particularly typical antipsychotics, have a dose-dependent effect on severe exacerbation of asthma. Analysis of the use of atypical antipsychotics did not show an increased risk of severe asthma exacerbation. Thus, we tentatively conclude that the use of typical antipsychotics is associated with a dose-dependent increased risk of severe asthma exacerbation.

We found that the use of typical antipsychotics led to a higher risk of severe asthma exacerbation (adjusted OR: 1.40), whereas the use of atypical antipsychotics did not. This finding is consistent with the different adverse events observed among typical and atypical antipsychotic users. The use of typical antipsychotics is more likely to cause extrapyramidal symptoms, and the use of atypical antipsychotics is often associated with weight gain and metabolic disturbance [25]. A possible explanation for the discordance of side effects between these two groups of drugs is that typical antipsychotics have a higher affinity to the dopaminergic receptor and lower affinity to the serotonin receptor compared to those for atypical agents [10]. The higher antipsychotic affinity to specific receptors is associated with a higher risk of different side effects [26].

We found that the simultaneous use of typical and atypical antipsychotics increases the risk of severe asthma exacerbation compared to the use of monotherapy. The combination of two antipsychotics is a widely used strategy for treatment-resistant schizophrenia [27], and a combination of typical and atypical antipsychotics is the most common management in real-world practice [28]. Compared with monotherapy, several studies have reported that combination therapy is associated with increased adverse events and mortality rates [29–32]. However, a recent meta-analysis did not show a different risk of serious adverse events between combination antipsychotics and monotherapy [33]. Further research is needed to investigate the risk of combination of atypical and atypical antipsychotics.

Antipsychotics block β2 adrenergic, M2 muscarinic, D1, and D2 dopaminergic receptors, which are found in human airway smooth muscle with the function of bronchial relaxation. Blocking these bronchodilation receptors increases airway smooth muscle tone and induces muscle spasms [21–24], causing severe asthma exacerbation. The results stratified by different receptor functions of antipsychotics in our study showed that there was a higher risk of severe asthma exacerbation for antipsychotics that function on the M2 muscarinic and D2 dopaminergic receptors. A plausible explanation is that most antipsychotics have higher affinities for the M2 muscarinic and D2 dopaminergic receptors than the β2 adrenergic receptor (Additional file 1: Table S4). Blocking the D2 dopaminergic receptor could also induce dystonia of the airway [34], causing acute exacerbation of airway disease.

Overgeneralization of our study results to patients with psychiatric disorder should be avoided. Poor controlling of psychiatric disorder increases the risk of asthma exacerbation. Patients with schizophrenia generally have low adherence to asthma treatment and adopt risky health behaviors, such as smoking [35]. The psychiatric disorder might affect patients’ perception and description of symptoms and coping skills, leading to poor asthma control. [19] In addition, depression may also be a trigger or consequence for patients with severe asthma [36]. In our multivariable analysis, we found that the adjusted ORs of schizophrenia is higher than antipsychotics (for antipsychotics, aOR:1.27, P-value:0.013; for schizophrenia, aOR: 5.46, P-value:0.011) (Additional file 1: Table S5), and use antipsychotics do not increase the risk of asthma severe exacerbation for patient with schizophrenia, bipolar or depression (Additional file 1: Table S6, Additional file 1: Table S7 and Additional file 1: Table S8). Based on the results above, use of antipsychotics might not increase risk of severe asthma exacerbation for patients with psychiatric disorder. Further studies for these patients are needed.

Our study had several limitations. First, the claims data did not include important information such as disease severity and pulmonary function data, and we did not consider different phenotypes of asthma. Nevertheless, we adjusted for the use of different inhaled bronchodilators or their combinations, which could be regarded as a surrogate for asthma severity. Consequently, the results were not significantly biased. Second, the diagnoses of asthma and its acute exacerbation should be based on medical history and physical examination instead of ICD codes only. Nevertheless, the accuracy of diagnostic records for asthma in the NHIRD has been validated [16]. We further defined newly diagnosed asthma patients as those who had more than one inpatient or two outpatient visits and excluded participants with the same diagnosis within the previous 2 years. Severe asthma exacerbation was defined as an acute exacerbation leading to ED visits or hospitalization in combination with the use of short-acting bronchodilators and systemic steroids. These strict definitions strengthen the validity of our results. Third, adherence to antipsychotics and other medications could not be confirmed using claims data. Nevertheless, our study used a case-crossover design, in which drug compliance during the case and control periods was assumed to be the same. We thought this factor did not significantly affect the results. Fourth, we did not include smoking status in the regression model because the NHIRD lacks information on smoking status. However, in our case-crossover study design, the time interval between the control period and the case period was 60 days, and the smoking status of each enrolled participant may not change in a short period. Approximately 70% of the enrolled patients in our study cohort were women. The smoking prevalence of women over 18 years of age in Taiwan was 3.9–5.5% during the study period [37]. In our study, the impact of smoking on severe asthma exacerbation might be small. Fifth, we cannot completely exclude the confounding by indication or other potential confounders by the sensitivity analyses. It should be cautious to overgeneralize our findings and further investigation for the effect of typical antipsychotics on severe asthma exacerbation is needed.

## Conclusions

In this nationwide population-based cohort study, patients with asthma showed a dose-dependent increase in the risk of severe asthma exacerbations when receiving typical antipsychotics. For patients with psychiatric disorders, antipsychotics might not increase the risk of severe asthma exacerbation. Further research for the effect of typical antipsychotics on severe asthma exacerbation is warranted.

## Supplementary Information

**Additional file 1. Table S1**. Elimination half-life of each antipsychotic. **Table S2**. Ninth and 10th revision international classification of diseases codes of comorbidity. **Table S3**. List of comedications in the presented study. **Table S4**. Receptor-binding profile of antipsychotics with bronchial relaxation effect. **Table S5**. The adjusted odds ratio of all covariates in multivariable conditional logistic regression. **Table S6**. Risk of severe asthma exacerbation by different classes of antipsychotics and doses in patients with schizophrenia. **Table S7**. Risk of severe asthma exacerbation by different classes of antipsychotics and doses in patients with depression. **Table S8**. Risk of severe asthma exacerbation by different classes of antipsychotics and doses in patients with bipolar disorder. **Table S9**. Risk of severe asthma exacerbation by different classes of antipsychotics and doses after excluding patients had psychiatric disorder related admission or ED visiting. **Table S10**. Risk of severe asthma exacerbation by different classes of antidepressants and doses.

## Data Availability

The de-linked datasets used and/or analysed during the current study are available from the corresponding author on reasonable request. The data are not publicly available because the use of the National Health Insurance Research Database is limited to research purposes only.

## Abbreviations

CI: Confidence interval
CM: Clinical manifestation
DDD: Defined daily dose
ED: Emergency department
ICD-9: International Classification of Diseases, Ninth Revision
NHIRD: National health insurance reimbursement database
OR: Odds ratio
SNRI: Serotonin–norepinephrine reuptake inhibitor
SSRI: Selective serotonin reuptake inhibitors
TCA: Tricyclic antidepressants

## References

[1] Papi A, Brightling C, Pedersen SE, Reddel HK (2018). Asthma. Lancet (London, England).

[2] Collaborators GCRD (2020). Prevalence and attributable health burden of chronic respiratory diseases, 1990–2017: a systematic analysis for the Global Burden of Disease Study 2017. Lancet Respir Med.

[3] Williams SA, Wagner S, Kannan H, Bolge SC (2009). The association between asthma control and health care utilization, work productivity loss and health-related quality of life. J Occup Environ Med.

[4] Ivanova JI, Bergman R, Birnbaum HG, Colice GL, Silverman RA, McLaurin K (2012). Effect of asthma exacerbations on health care costs among asthmatic patients with moderate and severe persistent asthma. J Allergy Clin Immunol.

[5] Miller MK, Lee JH, Miller DP, Wenzel SE (2007). Recent asthma exacerbations: a key predictor of future exacerbations. Respir Med.

[6] Chien IC, Hsu JH, Bih SH, Lin CH, Chou YJ, Lee CH, Chou P (2008). Prevalence, correlates, and disease patterns of antipsychotic use in Taiwan. Psychiatry Clin Neurosci.

[7] Mark TL (2010). For what diagnoses are psychotropic medications being prescribed? A nationally representative survey of physicians. CNS Drugs.

[8] O'Brien PL, Cummings N, Mark TL (2017). Off-label prescribing of psychotropic medication, 2005–2013: an examination of potential influences. Psychiatr Serv (Washington, DC).

[9] Carton L, Cottencin O, Lapeyre-Mestre M, Geoffroy PA, Favre J, Simon N, Bordet R, Rolland B (2015). Off-label prescribing of antipsychotics in adults, children and elderly individuals: a systematic review of recent prescription trends. Curr Pharm Des.

[10] Miyamoto S, Miyake N, Jarskog LF, Fleischhacker WW, Lieberman JA (2012). Pharmacological treatment of schizophrenia: a critical review of the pharmacology and clinical effects of current and future therapeutic agents. Mol Psychiatry.

[11] Siafis S, Tzachanis D, Samara M, Papazisis G (2018). Antipsychotic drugs: from receptor-binding profiles to metabolic side effects. Curr Neuropharmacol.

[12] Morales DR, Lipworth BJ, Donnan PT, Jackson C, Guthrie B (2017). Respiratory effect of beta-blockers in people with asthma and cardiovascular disease: population-based nested case control study. BMC Med.

[13] Crane J, Pearce N, Burgess C, Woodman K, Robson B, Beasley R (1992). Markers of risk of asthma death or readmission in the 12 months following a hospital admission for asthma. Int J Epidemiol.

[14] Mossman D, Lehrer DS (2000). Conventional and atypical antipsychotics and the evolving standard of care. Psychiatr Serv (Washington, DC).

[15] Wang JY, Yao TC, Tsai YT, Wu AC, Tsai HJ (2018). Increased dose and duration of statin use is associated with decreased asthma-related emergency department visits and hospitalizations. J Allergy Clin Immunol Pract.

[16] Su VY, Yang KY, Yang YH, Tsai YH, Perng DW, Su WJ, Chou KT, Su KC, Yen YF, Chen PC (2018). Use of ICS/LABA combinations or LAMA is associated with a lower risk of acute exacerbation in patients with coexistent COPD and asthma. J Allergy Clin Immunol Pract.

[17] Wang MT, Tsai CL, Lin CW, Yeh CB, Wang YH, Lin HL (2017). Association between antipsychotic agents and risk of acute respiratory failure in patients with chronic obstructive pulmonary disease. JAMA Psychiat.

[18] Wang MT, Lin CW, Tsai CL, Wang YH, Lai JH, Yeh CB, Huang YL, Hsu YJ (2020). Use of antipsychotics and the risk of acute respiratory failure among adults: a disease risk score-matched nested case-control study. Br J Clin Pharmacol.

[19] Baiardini I, Sicuro F, Balbi F, Canonica GW, Braido F (2015). Psychological aspects in asthma: do psychological factors affect asthma management?. Asthma Res Pract.

[20] Leucht S, Samara M, Heres S, Davis JM (2016). Dose equivalents for antipsychotic drugs: the DDD method. Schizophr Bull.

[21] Kotlikoff MI, Kamm KE (1996). Molecular mechanisms of beta-adrenergic relaxation of airway smooth muscle. Annu Rev Physiol.

[22] Fryer AD, Jacoby DB (1998). Muscarinic receptors and control of airway smooth muscle. Am J Respir Crit Care Med.

[23] Mizuta K, Zhang Y, Xu D, Mizuta F, D'Ovidio F, Masaki E, Emala CW (2013). The dopamine D1 receptor is expressed and facilitates relaxation in airway smooth muscle. Respir Res.

[24] Mizuta K, Zhang Y, Xu D, Masaki E, Panettieri RA, Emala CW (2012). The dopamine D(2) receptor is expressed and sensitizes adenylyl cyclase activity in airway smooth muscle. Am J Physiol Lung Cell Mol Physiol.

[25] Mathews M, Muzina DJ (2007). Atypical antipsychotics: new drugs, new challenges. Clevel Clin J Med.

[26] Olten B, Bloch MH (2018). Meta regression: Relationship between antipsychotic receptor binding profiles and side-effects. Prog Neuropsychopharmacol Biol Psychiatry.

[27] Dold M, Leucht S (2014). Pharmacotherapy of treatment-resistant schizophrenia: a clinical perspective. Evid Based Ment Health.

[28] Gallego JA, Bonetti J, Zhang J, Kane JM, Correll CU (2012). Prevalence and correlates of antipsychotic polypharmacy: a systematic review and meta-regression of global and regional trends from the 1970s to 2009. Schizophr Res.

[29] Gallego JA, Nielsen J, De Hert M, Kane JM, Correll CU (2012). Safety and tolerability of antipsychotic polypharmacy. Expert Opin Drug Saf.

[30] McIntyre RS, Jerrell JM (2008). Metabolic and cardiovascular adverse events associated with antipsychotic treatment in children and adolescents. Arch Pediatr Adolesc Med.

[31] Jerrell JM, McIntyre RS (2008). Adverse events in children and adolescents treated with antipsychotic medications. Hum Psychopharmacol.

[32] Joukamaa M, Heliövaara M, Knekt P, Aromaa A, Raitasalo R, Lehtinen V (2006). Schizophrenia, neuroleptic medication and mortality. Br J Psychiatry.

[33] Ortiz-Orendain J, Castiello-de Obeso S, Colunga-Lozano LE, Hu Y, Maayan N, Adams CE (2017). Antipsychotic combinations for schizophrenia. Cochrane Database Syst Rev.

[34] Yaginuma K, Watanabe M, Miyazaki K, Ono A, Murai H, Nodera M, Suzuki Y, Suyama K, Kawasaki Y, Hosoya M (2019). Haloperidol-induced dystonia due to sedation for upper gastrointestinal endoscopy: a pediatric case report. Case Rep Emerg Med.

[35] Joseph KS (1997). Asthma mortality and antipsychotic or sedative use. What is the link?. Drug Saf.

[36] Boulet LP (2009). Influence of comorbid conditions on asthma. Eur Respir J.

[37] Chiang CY, Chang HY (2016). A population study on the time trend of cigarette smoking, cessation, and exposure to secondhand smoking from 2001 to 2013 in Taiwan. Popul Health Metr.

