# Supplementary material for: Typical antipsychotics is associated with increased risk of severe exacerbation in asthma patients: a nationwide population-based cohort study
Source: BMC Pulm Med. 2022 Mar 14;22:85. doi: 10.1186/s12890-022-01883-6 (PMC8919619; doi:10.1186/s12890-022-01883-6)
Supplement: Supplementary file 1 — Additional file 1. Table S1. Elimination half-life of each antipsychotic. Table S2. Ninth and 10th revision international classification of diseases codes of comorbidity. Table S3. List of comedications in the presented study. Table S4. Receptor-binding profile of antipsychotics with bronchial relaxation effect. Table S5. The adjusted odds ratio of all covariates in multivariable conditional logistic regression. Table S6. Risk of severe asthma exacerbation by different classes of antipsychotics and doses in patients with schizophrenia. Table S7. Risk of severe asthma exacerbation by different classes of antipsychotics and doses in patients with depression. Table S8. Risk of severe asthma exacerbation by different classes of antipsychotics and doses in patients with bipolar disorder. Table S9. Risk of severe asthma exacerbation by different classes of antipsychotics and doses after excluding patients had psychiatric disorder related admission or ED visiting. Table S10. Risk of severe asthma exacerbation by different classes of antidepressants and doses. [file 12890_2022_1883_MOESM1_ESM.docx]

| **Supplementary Table 1.** **Elimination half-life of each antipsychotic**   \| Category \| Name of drug \| Elimination half life \| \| --- \| --- \| --- \| \| Atypical antipsychotics \| Amisulpride \| 4-5 hours^1^ \| \| Aripiprazole \| 75-146 hours^1^ \| \| Clozapine \| Parent: 8-12 hours^1^  Metabolites: (1) N-desmethylclozapine: 13.2 hours^1^  (2) N-oxide metabolite: 7 hours^1^ \| \| Olanzapine \| 30 hours^1^ \| \| Paliperidone \| 23 hours^1^ \| \| Quetiapine \| 6-7 hours^1^ \| \| Risperidone \| 3-20 hours^1^ \| \| Ziprasidone \| Ziprasidone hydrochloride: 7 hours^1^  Ziprasidone mesylate: 2-5 hours^1^ \| \| Zotepine \| Biexponential pharmacokinetics:  Alpha 0.9-1.3 hours^1^, beta 13.7-15.9 hours^1^  Metabolite: Norzotepine 12 hours^1^ \| \| Typical antipsychotics \| Chlorpromazine \| 6 hours^1^ \| \| Chlorprothixene \| Parent: 8-12 hours^1^  Metabolite:  Chlorprothixene sulfoxide 20-40 hours^1^ \| \| Flupentixol \| 35 hours \| \| Haloperidol \| Haloperidol lactate: 10-37 hours^1^ \| \| Levomepromazine \| Metabolite:  Methotrimeprazine sulfoxide 15 hours^1^ \| \| Loxapine \| 7.61 hours^1^ \| \| Perphenazine \| 9.5 hours^1^ \| \| Pimozide \| 55-66 hours^1^ \| \| Pipotiazine \| 11.2 hours^2^ \| \| Prochlorperazine \| 6.8-9 hours^1^ \| \| Sulpiride \| 6-8 hours^1^ \| \| Thioridazine \| 21-24 hours^1^ \| \| Trifluoperazine \| 24 hours^1^ \| \| Zuclopenthixol \| 20 hours^1^ \|   References:   1. In: Pharmacokinetics [database on the Internet] Greenwood Village (CO): Truven Health Analytics; [cited 2020 Aug 11]. Available from: www.micromedexsolutions.com. Subscription required to view. 2. De Schepper PJ, Vranckx C, Verbeeck R, Van den Berghe ML. Pipotiazine pharmacokinetics after p.o. and i.v. administration in man. Correlation between blood levels and effect on the handwriting area. Arzneimittelforschung. 1979;29(7):1056-1062.   **Supplementary Table 2. Ninth and 10^th^ revision international classification of diseases codes of comorbidity**   \| **Comorbidity** \| **Operative definition by ICD 9** \| **Operative definition by ICD 10** \| \| --- \| --- \| --- \| \| Stroke \| 430-438 \| I60-I64 \| \| Ischemic heart disease \| 410-414 \| I21-I25 \| \| Heart failure \| 428 \| I50 \| \| Depression \| 296.2x, 296.3x, 300.4x, 311.x \| F32-34 \| \| Anxiety \| 300, except 300.4 \| F40-45 \| \| Bipolar disorder \| 496.0, 496.1, 296.4-296.8 \| F30-31 \| \| Schizophrenia \| 295 \| F20-25, F28-29 \| \| Gastroesophageal reflux disease \| 530.11, 530.81 \| K21.0, K21.9 \| \| Obesity disorder \| 278.0x \| 278.0x \| \| Rhinitis, sinusitis \| 472.x, 473.x, 477.x \| J30.0-30.2, J30.81, J30.89, J30.9, J30.5,  J31, J32.0-32.4, J32.8-32.9, R09.82, \| \| Obstructive sleep apnea \| 327.23 \| G47.33 \| \| Chronic obstructive pulmonary disease \| 491.2, 493.2, 496 \| J44 \| \| Chronic bronchiolitis \| 491.0, 491.1, 491.8 \| J40, J41, J42 \| \| Emphysema \| 492 \| J43 \| \| Bronchiectasis \| 494 \| J47 \| \| Pneumonia \| 480-486, 487.0, 507 \| A37.91, A48.1, B25.0, J11.1, J11.00,  J11.08, J12-J18, J69, \| \| Acute upper airway infection \| 464, 465, 466 \| J04-J06, J20, J21 \|   **Supplementary Table 3. List of comedications in the presented study**   \| **Category** \| **Medication** \| \| --- \| --- \| \| **Cardiovascular medications** \| \| \| Antiplatelet \| Aspirin, clopidogrel, dipyridamole, ticagrelor, ticlopidine \| \| Cardiac selective β blocker \| Acebutolol, atenolol, betaxolol, bisoprolol, and metoprolol \| \| Non-selective β blocker \| Alprenolol, carteolol, carvedilol, labetalol, pindolol, propranolol, timolol \| \| Statins \| Atorvastatin, fluvastatin, lovastatin, pitavastatin, pravastatin, rosuvastatin, and simvastatin \| \| **Anti-inflammation agents** \| \| \| NSAID \| Aceclofenac, acemetacin, alclofenac, benzydamine, celecoxib, diclofenac, ethenzamide, etodolac, etofenamate, etoricoxib, fenbufen, flufenamic acid, flurbiprofen, ibuprofen, indomethacin, ketoprofen, ketorolac, meclofenamic acid, mefenamic acid, meloxicam, mepirizole, nabumetone, naproxen, nefopam, niflumic acid, nimesulide, phenylbutazone, piroxicam, salsalate, sulindac, tenoxicam, tiaprofenic acid, tiaramide, tolfenamic acid, and tolmetin \| \| Others \| Colchicine, metformin \| \| **Psychoactive drugs** \| \| \| Typical antipsychotics \| Chlorpromazine, chlorprothixene, clopenthixol, flupentixol, haloperidol, levomepromazine, loxapine, perphenazine, pimozide, pipotiazine, prochlorperazine, sulpiride thioridazine, trifluoperazine \| \| Atypical antipsychotics \| Amisulpride, aripiprazole, clozapine, olanzapine, paliperidone, quetiapine, risperidone, ziprasidone, zotepine \| \| Antidepressants \| Amitriptyline, bupropion, citalopram, clomipramine, dothiepin, doxepin, duloxetine, escitalopram, fluoxetine, fluvoxamine, imipramine, maprotiline, melitracen, milnacipran, mirtazapine, moclobemide, paroxetine, sertraline, trazodone, venlafaxine \| \| Lithium \| Lithium carbonate \| \| **Anticholinergic agents** \| \| \| Bladder antimuscarinics \| Flavoxate, oxybutynin, solifenacin, tolterodine, and trospium \| \| Gastrointestinal antispasmodics \| Alverine, atropine, dicyclomine, dicycloverine, glycopyrrolate, homatropine, mebeverine, mepenzolate, methscopolamine, otilonium, piperidolate, propantheline, scopolamine, timepidium, valethamate \| \| Antiparkinsonian \| Benztropine, biperiden, piroheptine and trihexyphenidyl \| \| **Antihistamines** \| \| \| First generation \| Brompheniramine, buclizine, chlorcyclizine, chlorpheniramine, clemastine, cyclizine, cyproheptadine, dexchlorpheniramine, dimenhydrinate, diphenhydramine, homochlorcyclizine, hydroxyzine, ketotifen, meclizine, mequitazine, promethazine, triprolidine \| \| Second/third generation \| Astemizole, cetirizine, desloratadine, ebastine, fexofenadine, levocetirizine, Loratadine, mebhydroline, mizolastine, terfenadine \| \| **Bronchodilators** \| \| \| **Long-acting bronchodilators** \| \| \| ICS \| Beclomethasone dipropionate, budesonide, ciclesonide, fluticasone propionate. \| \| LABA \| Indacaterol. \| \| LAMA \| Tiotropium, umeclidinium. \| \| LABA + LAMA \| Indacaterol plus glycopyrronium; Vilanterol plus umeclidinium; Olodacterol plus tiotropium. \| \| LABA + ICS \| Formoterol plus beclomethasone, formoterol plus budesonide, salmeterol plus fluticasone; vilanterol plus fluticasone furoate; fluticasone plus formoterol. \| \| ICS + LABA + LAMA \| Fluticasone plus umeclidinium plus vilanterol. \| \| **Short-acting bronchodilators** \| \| \| SABA \| Fenoterol, salbutamol, terbutaline. \| \| SAMA \| Ipratropium bromide. \| \| SABA + SAMA \| Fenoterol plus ipratropium bromide, salbutamol plus ipratropium bromide. \| \| **Oral bronchodilator** \| \| \| Xanthine inhibitor \| Aminophylline, theophylline \| \| Leukotriene inhibitor \| Montelukast, zafirlukast \| \| **Systemic corticosteroid** \| \| \| Oral corticosteroid \| Betamethasone, budesonide, cortisone, dexamethasone, methylprednisolone, fludrocortisone, prednisolone, triamcinolone. \| \| Intravenous corticosteroid \| Betamethasone, dexamethasone, hydrocortisone, methylprednisolone, prednisolone, triamcinolone \|   ICS: inhaled corticosteroid, LABA: long-acting beta-agonist; LAMA: long-acting muscarinic antagonist; NSAID: non-steroidal anti-inflammatory drug; SABA: short-acting beta-agonist; SAMA: short-acting muscarinic antagonist.   \| Anti-psychotics^*^ \| β2 adrenergic receptor \| Muscarinic 2 receptor \| Dopamine 1 receptor \| Dopamine 2 receptor \| \| --- \| --- \| --- \| --- \| --- \| \| Amisulpride \| - \| - \| - \| +++ \| \| Aripiprazole \| + \| - \| + (partial agonist) ^**^ \| +++ (partial agonist) ^**^ \| \| Chlorpromazine \| - \| + \| ++ \| +++ \| \| Chlorprothixene \| NA \| ++ \| ++ \| +++ \| \| Clopenthixol \| NA \| NA \| NA \| NA \| \| Clothiapine \| NA \| NA \| NA \| NA \| \| Clozapine \| - \| ++ \| + \| + \| \| Droperidol \| NA \| NA \| NA \| ++++ \| \| Flupentixol \| NA \| NA \| NA \| NA \| \| Fluphenazine \| - \| - \| ++ \| ++++ \| \| Haloperidol \| - \| - \| ++ \| +++ \| \| Levomepromazine \| NA \| NA \| NA \| NA \| \| Loxapine \| - \| + \| ++ \| ++ \| \| Olanzapine \| - \| ++ \| ++ \| ++ \| \| Paliperidone \| NA \| NA \| + \| +++ \| \| Perphenazine \| NA \| - \| - \| ++++ \| \| Pimozide \| NA \| NA \| - \| +++ \| \| Pipotazine \| NA \| NA \| NA \| NA \| \| Prochlorperazine \| NA \| - \| NA \| +++ \| \| Quetiapine \| - \| + \| + \| + \| \| Risperidone \| - \| - \| + \| +++ \| \| Sulpiride \| NA \| NA \| - \| ++ \| \| Thioridazine \| - \| + \| ++ \| ++ \| \| Trifluoperazine \| NA \| NA \| NA \| +++ \| \| Ziprasidone \| - \| - \| + \| +++ \| \| Zotepine \| - \| + \| ++ \| +++ \| \| Zuclopenthixol \| NA \| NA \| NA \| NA \|   **Supplementary Table 4. Receptor-binding profile of antipsychotics with bronchial relaxation effect**  NA: not available.  ^*^ The number of crosses are correlated to binding affinity. Ki > 1000: -; 100 < Ki < 1000: +; 10 < Ki < 100: ++; 1 < Ki < 10: +++; 1 < Ki: ++++. Note: Ki values were retrieved from table of *Current Neuropharmacology.* 2018;16:1210-1223 and National Institute of Mental Health’s Psychoactive Drug Screening Program database: http://kidbdev.med.unc.edu/databases/pdsp.php (accessed 2020/08), based on Roth BL, *et al.* Screening the receptorome to discover the molecular targets for plant-derived psychoactive compounds: a novel approach for CNS drug discovery. *Pharmacol Ther.* 2004;102:99–11.  **^**^** We did not analyze dopamine receptor affinity of Aripiprazole because its partial agonist and antagonist effect.  **Supplementary Table 5.** **The adjusted odds ratio of all covariates in multivariable conditional logistic regression.**   \|  \| Conditional logistic regression \| \| \| --- \| --- \| --- \| \| Characteristics \| Adjusted OR (95% CI) \| *P* value \| \| Comorbidities: \|  \|  \| \| Heart failure \| 1.68 (1.11-2.54) \| 0.015 \| \| Ischemic heart disease \| 1.35 (1.02-1.79) \| 0.039 \| \| Stroke \| 1.41 (0.96-2.07) \| 0.077 \| \| GERD \| 1.59 (1.27-2.00) \| < 0.001 \| \| Obesity \| 3.98 (1.87-8.45) \| < 0.001 \| \| Rhinosinusitis \| 1.15 (1.02-1.28) \| 0.020 \| \| Psychiatric disorder \|  \|  \| \| Schizophrenia \| 5.46 (1.48-20.18) \| 0.011 \| \| Respiratory infection \|  \|  \| \| Pneumonia \| 1.82 (1.45-2.27) \| < 0.001 \| \| Acute upper airway infection \| 1.48 (1.37-1.60) \| < 0.001 \| \| Medications: \|  \|  \| \| Cardiovascular drugs \|  \|  \| \| Antiplatelet \| 1.33 (1.13-1.56) \| 0.001 \| \| Cardioselective β-blockers \| 1.09 (0.90-1.33) \| 0.386 \| \| Non-selective β-blockers \| 1.26 (1.05-1.51) \| 0.014 \| \| Anti-inflammation \|  \|  \| \| NSAID \| 1.82 (1.71-1.94) \| <0.001 \| \| Psychoactive drugs \|  \|  \| \| Antidepressants \| 1.15 (0.96-1.38) \| 0.125 \| \| Antipsychotics \| 1.27 (1.05-1.54) \| 0.013 \| \| Anticholinergic agents \|  \|  \| \| Bladder \| 1.27 (0.86-1.86) \| 0.225 \| \| Gastrointestinal tract \| 1.40 (1.26-1.56) \| < 0.001 \| \| Antihistamine \|  \|  \| \| First generation \| 1.46 (1.35-1.57) \| < 0.001 \| \| Second/third generation \| 1.47 (1.37-1.57) \| < 0.001 \| \| Inhaled or oral bronchodilators \|  \|  \| \| SABA \| 1.86 (1.69-2.05) \| < 0.001 \| \| SAMA \| 2.50 (1.78-3.53) \| < 0.001 \| \| SABA + SAMA \| 1.62 (1.14-2.31) \| 0.008 \| \| LABA + ICS \| 0.92 (0.82-1.03) \| 0.156 \| \| ICS \| 0.88 (0.72-1.08) \| 0.217 \| \| Xanthium \| 2.18 (2.01-2.38) \| < 0.001 \| \| Leukotriene receptor antagonist \| 1.07 (0.87-1.30) \| 0.522 \|   GERD: Gastroesophageal reflux disease; ICS: inhaled corticosteroid, LABA: long-acting beta-agonist; NSAID: non-steroidal anti-inflammatory drug; OR: odds ratio; SABA: short-acting beta-agonist; SAMA: short-acting muscarinic antagonist. |
| --- | --- | --- | --- | --- | --- | --- | --- | --- | --- | --- | --- | --- | --- | --- | --- | --- | --- | --- | --- | --- | --- | --- | --- | --- | --- | --- | --- | --- | --- | --- | --- | --- | --- | --- | --- | --- | --- | --- | --- | --- | --- | --- | --- | --- | --- | --- | --- | --- | --- | --- | --- | --- | --- | --- | --- | --- | --- | --- | --- | --- | --- | --- | --- | --- | --- | --- | --- | --- | --- | --- | --- | --- | --- | --- | --- | --- | --- | --- | --- | --- | --- | --- | --- | --- | --- | --- | --- | --- | --- | --- | --- | --- | --- | --- | --- | --- | --- | --- | --- | --- | --- | --- | --- | --- | --- | --- | --- | --- | --- | --- | --- | --- | --- | --- | --- | --- | --- | --- | --- | --- | --- | --- | --- | --- | --- | --- | --- | --- | --- | --- | --- | --- | --- | --- | --- | --- | --- | --- | --- | --- | --- | --- | --- | --- | --- | --- | --- | --- | --- | --- | --- | --- | --- | --- | --- | --- | --- | --- | --- | --- | --- | --- | --- | --- | --- | --- | --- | --- | --- | --- | --- | --- | --- | --- | --- | --- | --- | --- | --- | --- | --- | --- | --- | --- | --- | --- | --- | --- | --- | --- | --- | --- | --- | --- | --- | --- | --- | --- | --- | --- | --- | --- | --- | --- | --- | --- | --- | --- | --- | --- | --- | --- | --- | --- | --- | --- | --- | --- | --- | --- | --- | --- | --- | --- | --- | --- | --- | --- | --- | --- | --- | --- | --- | --- | --- | --- | --- | --- | --- | --- | --- | --- | --- | --- | --- | --- | --- | --- | --- | --- | --- | --- | --- | --- | --- | --- | --- | --- | --- | --- | --- | --- | --- | --- | --- | --- | --- | --- | --- | --- | --- | --- | --- | --- | --- | --- | --- | --- | --- | --- | --- | --- | --- | --- | --- | --- | --- | --- | --- | --- | --- | --- | --- | --- | --- | --- | --- | --- | --- | --- | --- | --- | --- | --- | --- | --- | --- | --- | --- | --- | --- | --- | --- | --- | --- | --- | --- | --- | --- | --- | --- | --- | --- | --- | --- | --- | --- | --- | --- | --- | --- | --- | --- | --- | --- | --- | --- | --- | --- | --- | --- | --- | --- | --- | --- | --- | --- | --- | --- | --- | --- | --- | --- | --- | --- | --- | --- | --- | --- | --- | --- | --- | --- | --- | --- | --- | --- | --- | --- | --- | --- | --- | --- | --- | --- | --- | --- | --- | --- | --- | --- | --- | --- | --- | --- | --- | --- | --- | --- | --- | --- | --- | --- | --- | --- | --- | --- | --- | --- | --- | --- | --- | --- | --- | --- | --- | --- | --- | --- | --- | --- | --- | --- | --- | --- | --- | --- | --- | --- | --- | --- | --- | --- | --- | --- | --- | --- | --- | --- | --- | --- | --- | --- | --- | --- | --- | --- |

**Supplementary Table 6.** Risk of severe asthma exacerbation by different classes of antipsychotics and doses in patients with schizophrenia

|  | No. (%) | |  | Conditional logistic regression | | | |
| --- | --- | --- | --- | --- | --- | --- | --- |
| Characteristics | Case period  (n=151) | Control period  (n=151) |  | Crude OR  (95% CI) | P value | Adjusted OR ^a^  (95% CI) | P value |
| No use of antipsychotics | 44 (29.14) | 48 (31.79) |  | Ref. |  | Ref. |  |
| Any use of antipsychotics | 107 (70.86) | 103 (68.21) |  | 1.24 (0.65-2.34) | 0.517 | 1.44 (0.59-3.51) | 0.423 |
| Antipsychotics class ^b^ |  |  |  |  |  |  |  |
| Typical only | 9 (5.96) | 7 (4.64) |  | 4.02 (0.44-36.56) | 0.217 | – | – |
| Atypical only | 85 (56.29) | 92 (60.93) |  | 1.02 (0.52-1.99) | 0.973 | 1.30 (0.50-3.40) | 0.589 |
| Both | 13 (8.61) | 4 (2.65) |  | – | – | – | – |
| Dose |  |  |  |  |  |  |  |
| Low (≤0.25 DDD) | 69 (45.70) | 63 (41.72) |  | 1.29 (0.66-2.52) | 0.451 | 1.46 (0.59-3.64) | 0.412 |
| Medium to high (>0.25 DDD) | 38 (25.17) | 40 (26.49) |  | 1.13 (0.54-2.36) | 0.738 | 1.52 (0.56-4.17) | 0.412 |
| Dose (for Typical only) |  |  |  |  |  |  |  |
| Low (≤0.25 DDD) | 6 (11.32) | 3 (5.45) |  | 3.00 (0.20-44.86) | 0.426 | 1.46 (0.06-33.41) | 0.814 |
| Medium to high (>0.25 DDD) | 3 (5.66) | 4 (7.27) |  | 1.00 (0.05-19.36) | 1.000 | 0.55 (0.02-15.06) | 0.721 |
| Dose (for Atypical only) |  |  |  |  |  |  |  |
| Low (≤0.25 DDD) | 55 (42.64) | 59 (42.14) |  | 1.07 (0.52-2.19) | 0.855 | 1.34 (0.48-3.75) | 0.579 |
| Medium to high (>0.25 DDD) | 30 (23.26) | 33 (23.57) |  | 1.05 (0.48-2.29) | 0.900 | 1.43 (0.46-4.42) | 0.534 |

DDD: defined daily dose; OR: odds ratio.

^a^ Adjusted for heart failure, ischemic heart disease, stroke, gastroesophageal reflux disease, obesity disorder, rhinosinusitis, depression, bipolar disorder, pneumonia, acute upper airway infection, antiplatelet agents, cardioselective β-blocker, non-selective β-blocker, NSAID, anti-psychotics, anti-depressants, bladder anticholinergic agents, gastrointestinal tract anticholinergic agents, first generation anti-histamine, second/third generation anti-histamine, short-acting beta-agonist, short-acting muscarinic antagonist, short-acting beta-agonist plus short-acting muscarinic antagonist, long-acting beta-agonist plus long-acting beta-agonist, long-acting beta-agonist, xanthine inhibitor, leukotriene receptor antagonist.

^b^ See Supplementary Table 1.

Note: Crude and adjusted OR of both antipsychotics use, adjusted OR of typical antipsychotics use were not available due to the small sample size.

**Supplementary Table 7.** Risk of severe asthma exacerbation by different classes of antipsychotics and doses in patients with depression

|  | No. (%) | |  | Conditional logistic regression | | | |
| --- | --- | --- | --- | --- | --- | --- | --- |
| Characteristics | Case period  (n=823) | Control period  (n=823) |  | Crude OR  (95% CI) | P value | Adjusted OR ^a^  (95% CI) | P value |
| No use of antipsychotics | 221 (26.85) | 207 (25.15) |  | Ref. |  | Ref. |  |
| Any use of antipsychotics | 602 (73.15) | 616 (74.85) |  | 1.22 (0.88-1.70) | 0.241 | 1.35 (0.90-2.03) | 0.145 |
| Antipsychotics class ^b^ |  |  |  |  |  |  |  |
| Typical only | 54 (6.56) | 49 (5.95) |  | 1.19 (0.72-1.95) | 0.501 | 1.33 (0.75-2.35) | 0.331 |
| Atypical only | 157 (19.08) | 147 (17.86) |  | 1.26 (0.82-1.94) | 0.294 | 1.39 (0.83-2.34) | 0.214 |
| Both | 10 (1.22) | 11 (1.34) |  | 0.97 (0.29-3.27) | 0.954 | 1.05 (0.27-4.09) | 0.939 |
| Dose |  |  |  |  |  |  |  |
| Low (≤0.25 DDD) | 172 (20.90) | 166 (20.17) |  | 1.19 (0.85-1.66) | 0.317 | 1.30 (0.86-1.96) | 0.212 |
| Medium to high (>0.25 DDD) | 49 (5.95) | 41 (4.98) |  | 1.48 (0.84-2.61) | 0.175 | 1.99 (1.02-3.87) | 0.044 |
| Dose (for Typical only) |  |  |  |  |  |  |  |
| Low (≤0.25 DDD) | 49 (7.47) | 46 (6.92) |  | 1.06 (0.64-1.76) | 0.829 | 1.07 (0.60-1.92) | 0.819 |
| Medium to high (>0.25 DDD) | 5 (0.76) | 3 (0.45) |  | 2.10 (0.36-12.05) | 0.407 | 4.75 (0.67-33.82) | 0.120 |
| Dose (for Atypical only) |  |  |  |  |  |  |  |
| Low (≤0.25 DDD) | 116 (15.28) | 115 (15.07) |  | 1.29 (0.82-2.05) | 0.276 | 1.43 (0.82-2.49) | 0.211 |
| Medium to high (>0.25 DDD) | 41 (5.40) | 32 (4.19) |  | 1.72 (0.90-3.28) | 0.104 | 2.19 (1.01-4.75) | 0.048 |

DDD: defined daily dose; OR: odds ratio.

^a^ Adjusted for heart failure, ischemic heart disease, stroke, gastroesophageal reflux disease, obesity disorder, rhinosinusitis, schizophrenia, bipolar disorder, pneumonia, acute upper airway infection, antiplatelet agents, cardioselective β-blocker, non-selective β-blocker, NSAID, anti-psychotics, anti-depressants, bladder anticholinergic agents, gastrointestinal tract anticholinergic agents, first generation anti-histamine, second/third generation anti-histamine, short-acting beta-agonist, short-acting muscarinic antagonist, short-acting beta-agonist plus short-acting muscarinic antagonist, long-acting beta-agonist plus long-acting beta-agonist, long-acting beta-agonist, xanthine inhibitor, leukotriene receptor antagonist.

^b^ See Supplementary Table 1.

**Supplementary Table 8.** Risk of severe asthma exacerbation by different classes of antipsychotics and doses in patients with bipolar disorder

|  | No. (%) | |  | Conditional logistic regression | | | |
| --- | --- | --- | --- | --- | --- | --- | --- |
| Characteristics | Case period  (n=167) | Control period  (n=167) |  | Crude OR  (95% CI) | P value | Adjusted OR ^a^  (95% CI) | P value |
| No use of antipsychotics | 77 (46.11) | 75 (44.91) |  | Ref. |  | Ref. |  |
| Any use of antipsychotics | 90 (53.89) | 92 (55.09) |  | 0.91 (0.50-1.67) | 0.758 | 0.75 (0.33-1.72) | 0.500 |
| Antipsychotics class ^b^ |  |  |  |  |  |  |  |
| Typical only | 9 (5.39) | 9 (5.39) |  | 0.98 (0.20-4.89) | 0.983 | 1.28 (0.19-8.54) | 0.801 |
| Atypical only | 76 (45.51) | 80 (47.90) |  | 0.90 (0.48-1.70) | 0.745 | 0.66 (0.28-1.58) | 0.352 |
| Both | 5 (2.99) | 3 (1.80) |  | 2.70 (0.26-28.34) | 0.408 | 2.78 (0.22-34.96) | 0.428 |
| Dose |  |  |  |  |  |  |  |
| Low (≤0.25 DDD) | 68 (40.72) | 73 (43.71) |  | 0.87 (0.47-1.62) | 0.654 | 0.72 (0.30-1.69) | 0.445 |
| Medium to high (>0.25 DDD) | 22 (13.17) | 19 (11.38) |  | 1.11 (0.47-2.62) | 0.811 | 0.87 (0.30-2.47) | 0.787 |
| Dose (for Typical only) |  |  |  |  |  |  |  |
| Low (≤0.25 DDD) | 9 (10.47) | 9 (10.71) |  | 0.67 (0.11-3.99) | 0.657 | 1.36 (0.04-44.97) | 0.863 |
| Medium to high (>0.25 DDD) | 0 (0.00) | 0 (0.00) |  | – | – | – | – |
| Dose (for Atypical only) |  |  |  |  |  |  |  |
| Low (≤0.25 DDD) | 56 (36.60) | 62 (40.00) |  | 0.91 (0.47-1.78) | 0.790 | 0.50 (0.19-1.32) | 0.160 |
| Medium to high (>0.25 DDD) | 20 (13.07) | 18 (11.61) |  | 1.08 (0.45-2.61) | 0.862 | 0.59 (0.19-1.82) | 0.360 |

DDD: defined daily dose; OR: odds ratio.

^a^ Adjusted for heart failure, ischemic heart disease, stroke, gastroesophageal reflux disease, obesity disorder, rhinosinusitis, depression, pneumonia, acute upper airway infection, antiplatelet agents, cardioselective β-blocker, non-selective β-blocker, NSAID, anti-psychotics, anti-depressants, bladder anticholinergic agents, gastrointestinal tract anticholinergic agents, first generation anti-histamine, second/third generation anti-histamine, short-acting beta-agonist, short-acting muscarinic antagonist, short-acting beta-agonist plus short-acting muscarinic antagonist, long-acting beta-agonist plus long-acting beta-agonist, long-acting beta-agonist, xanthine inhibitor, leukotriene receptor antagonist.

^b^ See Supplementary Table 1.

**Supplementary Table 9**. Risk of severe asthma exacerbation by different classes of antipsychotics and doses after excluding patients had psychiatric disorder related admission or ED visiting ^a^

|  | No. (%) | |  | Conditional logistic regression | | | |  |
| --- | --- | --- | --- | --- | --- | --- | --- | --- |
| Characteristics | Case period  (n=18573) | Control period  (n=18573) |  | Crude OR  (95% CI) | P value | Adjusted OR ^a^  (95% CI) | P value | P for trend |
| No use of antipsychotics | 17908 | 18017 |  | Ref. |  | Ref. |  |  |
| Any use of antipsychotics | 665 | 556 |  | 1.49 (1.26-1.76) | <0.001 | 1.29 (1.06-1.56) | 0.009 |  |
| Antipsychotics class |  |  |  |  |  |  |  |  |
| Typical only | 263 | 190 |  | 1.61 (1.29-2.02) | <0.001 | 1.39 (1.08-1.78) | 0.010 |  |
| Atypical only | 375 | 350 |  | 1.29 (1.00-1.66) | 0.051 | 1.13 (0.86-1.50) | 0.386 |  |
| Both | 27 | 16 |  | 2.69 (1.17-6.18) | 0.020 | 2.37 (1.01-5.56) | 0.047 |  |
| Dose |  |  |  |  |  |  |  |  |
| Low (≤0.25 DDD) | 567 | 474 |  | 1.47 (1.24-1.75) | <0.001 | 1.27 (1.05-1.54) | 0.015 | 0.012 |
| Medium to high (>0.25 DDD) | 98 | 82 |  | 1.71 (1.19-2.47) | 0.004 | 1.67 (1.12-2.49) | 0.012 |  |
| Dose (for Typical) |  |  |  |  |  |  |  |  |
| Low (≤0.25 DDD) | 249 | 181 |  | 1.59 (1.27-2.01) | <0.001 | 1.36 (1.06-1.75) | 0.016 | 0.006 |
| Medium to high (>0.25 DDD) | 14 | 9 |  | 2.84 90.95-8.46) | 0.061 | 4.49 (1.35-14.89) | 0.014 |  |
| Dose (for Atypical) |  |  |  |  |  |  |  |  |
| Low (≤0.25 DDD) | 299 | 285 |  | 1.29 (0.99-1.68) | 0.059 | 1.12 (0.83-1.50) | 0.457 | 0.359 |
| Medium to high (>0.25 DDD) | 76 | 65 |  | 1.52 (0.99-2.33) | 0.057 | 1.41 (0.88-2.25) | 0.153 |  |

DDD: defined daily dose, OR: odds ratio

^a^ Psychiatric disorder related admission or ED visiting were defined as least one depression, bipolar disorder, or schizophrenia-related admissions or emergency department visits within 180 days prior to the case and control periods, and patients had to receive psychoactive drugs (**Supplementary Table 3**) in the first day of admission or ED visit.

^b^ Adjusted for heart failure, ischemic heart disease, stroke, gastroesophageal reflux disease, obesity disorder, rhinosinusitis, schizophrenia, pneumonia, acute upper airway infection, antiplatelet agents, cardioselective β-blocker, non-selective β-blocker, NSAID, anti-psychotics, anti-depressants, bladder anticholinergic agents, gastrointestinal tract anticholinergic agents, first generation anti-histamine, second/third generation anti-histamine, short-acting beta-agonist, short-acting muscarinic antagonist, short-acting beta-agonist plus short-acting muscarinic antagonist, long-acting beta-agonist plus long-acting beta-agonist, long-acting beta-agonist, xanthine inhibitor, leukotriene receptor antagonist.

**Supplementary Table 10.** Risk of severe asthma exacerbation by different classes of antidepressants and doses

|  | No. (%) | |  | Conditional logistic regression | | | |
| --- | --- | --- | --- | --- | --- | --- | --- |
| Characteristics | Case period  (n=18351) | Control period  (n=18387) |  | Crude OR  (95% CI) | P value | Adjusted OR ^a^  (95% CI) | P value |
| No use of antidepressants | 17700 (96.45) | 17800 (96.81) |  | Ref. |  | Ref. |  |
| Any use of antidepressants | 651 (3.55) | 587 (3.19) |  | 1.41 (1.16-1.71) | 0.001 | 1.15 (0.96-1.38) | 0.140 |
| Anti-depressants class |  |  |  |  |  |  |  |
| SSRI/SNRI only | 502 (2.74) | 455 (2.47) |  | 1.42 (1.12-1.79) | 0.004 | 1.26 (0.97-1.64) | 0.081 |
| TCA only | 134 (0.73) | 112 (0.61) |  | 1.44 (1.03-2.02) | 0.034 | 1.27 (0.88-1.85) | 0.207 |
| Both | 15 (0.08) | 20 (0.11) |  | 0.53 (0.19-1.53) | 0.240 | 0.55 (0.17-1.75) | 0.309 |
| Dose |  |  |  |  |  |  |  |
| Low (≤0.25 DDD) | 119 (0.65) | 100 (0.54) |  | 1.38 (1.02-1.87) | 0.039 | 1.22 (0.87-1.71) | 0.245 |
| Medium to high (>0.25 DDD) | 532 (2.90) | 487 (2.65) |  | 1.42 (1.14-1.78) | 0.002 | 1.27 (0.99-1.62) | 0.062 |
| Dose (for SSRI/SNRI only) |  |  |  |  |  |  |  |
| Low (≤0.25 DDD) | 33 (0.18) | 31 (0.17) |  | 1.25 (0.71-2.17) | 0.441 | 1.07 (0.57-1.98) | 0.841 |
| Medium to high (>0.25 DDD) | 469 (2.58) | 424 (2.32) |  | 1.40 (1.09-1.79) | 0.008 | 1.27 (0.97-1.67) | 0.088 |
| Dose (for TCA only) |  |  |  |  |  |  |  |
| Low (≤0.25 DDD) | 85 (0.48) | 67 (0.37) |  | 1.52 (1.04-2.22) | 0.029 | 1.34 (0.88-2.03) | 0.169 |
| Medium to high (>0.25 DDD) | 49 (0.27) | 45 (0.25) |  | 1.47 (0.84-2.60) | 0.182 | 1.19 (0.63-2.23) | 0.599 |

DDD: defined daily dose; OR: odds ratio; SNRI: Serotonin–norepinephrine reuptake inhibitor; SSRI: Selective serotonin reuptake inhibitors; TCA: Tricyclic antidepressants

^a^ Adjusted for heart failure, ischemic heart disease, stroke, gastroesophageal reflux disease, obesity disorder, rhinosinusitis, schizophrenia, depression, bipolar disorder, pneumonia, acute upper airway infection, antiplatelet agents, cardioselective β-blocker, non-selective β-blocker, NSAID, anti-psychotics, anti-depressants, bladder anticholinergic agents, gastrointestinal tract anticholinergic agents, first generation anti-histamine, second/third generation anti-histamine, short-acting beta-agonist, short-acting muscarinic antagonist, short-acting beta-agonist plus short-acting muscarinic antagonist, long-acting beta-agonist plus long-acting beta-agonist, long-acting beta-agonist, xanthine inhibitor, leukotriene receptor antagonist.
